# Dermoscopy of Kindler Syndrome

**DOI:** 10.5826/dpc.1002a34

**Published:** 2020-04-03

**Authors:** Shekhar Neema, Preema Sinha, Sunmeet Sandhu, Sweta Mukherjee, S. Radhakrishnan

**Affiliations:** 1 Department of Dermatology, Armed Forces Medical College, Pune, India; 2 Department of Pediatrics, Command Hospital, Pune, India

**Keywords:** Kindler syndrome, dermoscopy, genodermatoses

## Introduction

Kindler syndrome is a rare autosomal recessive genodermatosis characterized by acral bullae in infancy followed by photosensitivity and progressive poikiloderma. The gene involved in Kindler syndrome is *FERMT1*, which encodes ferritin family homolog 1 protein linking actin to the underlying extracellular matrix.

## Case Presentation

**Figure 1:**
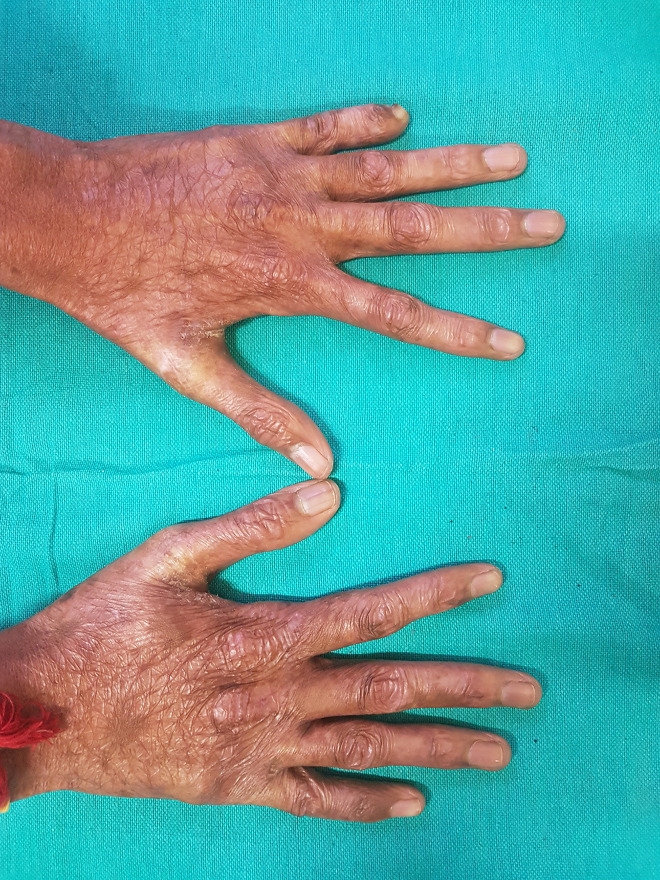
Atrophy of skin of dorsum of both hands (cigarette paper scarring).

**Figure 2:**
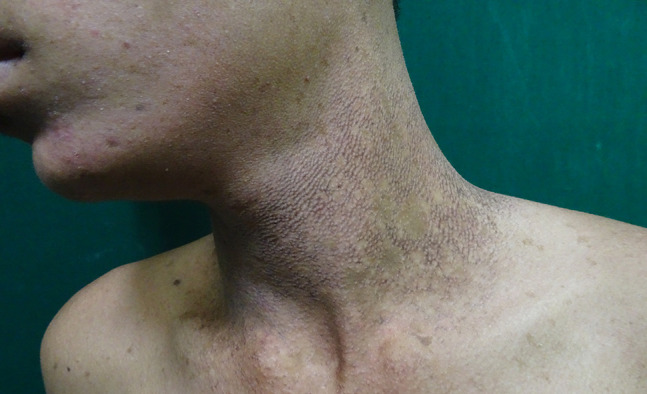
Poikiloderma (atrophy, telangiectasia, and hyperpigmentation) involving the neck.

**Figure 3:**
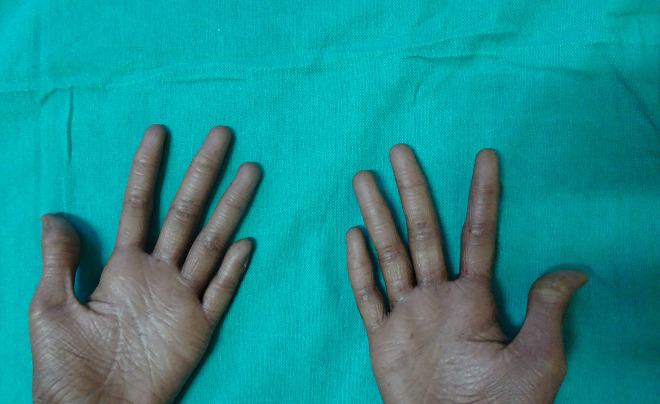
Waxy keratoderma involving both palms.

**Figure 4:**
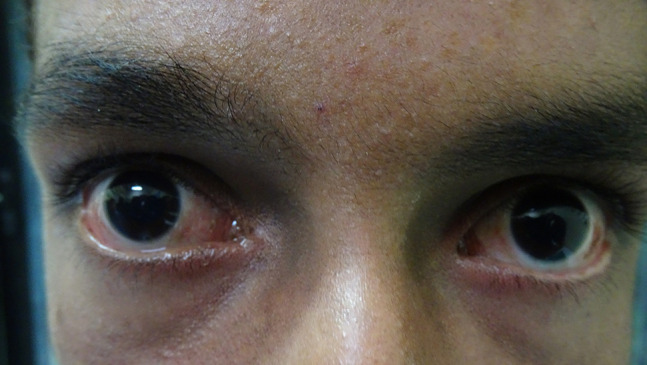
Conjunctivitis.

**Figure 5:**
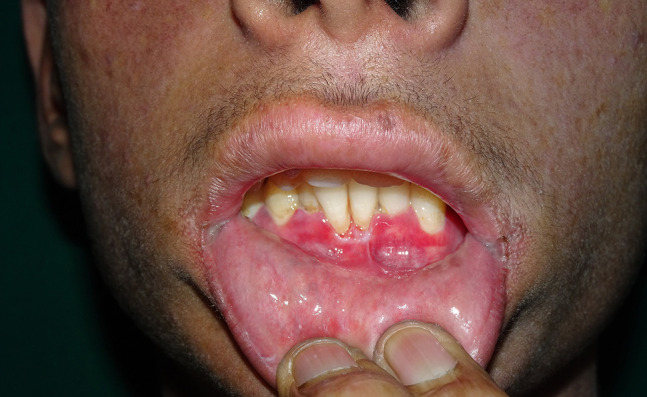
Periodontitis.

**Figure 6:**
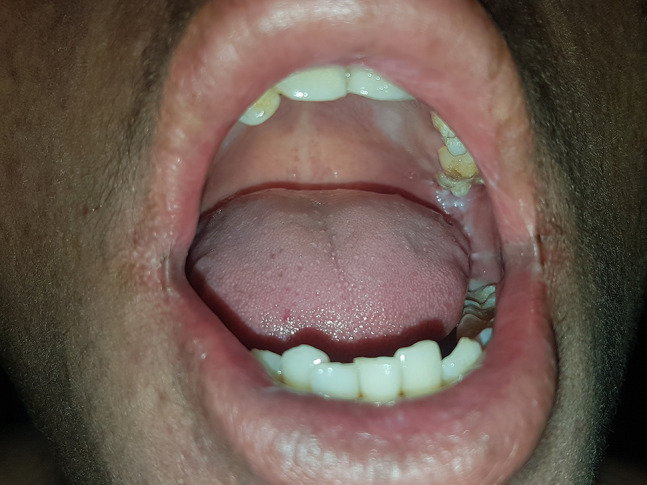
Oral leukokeratosis involving gingival mucosa.

**Figure 7:**
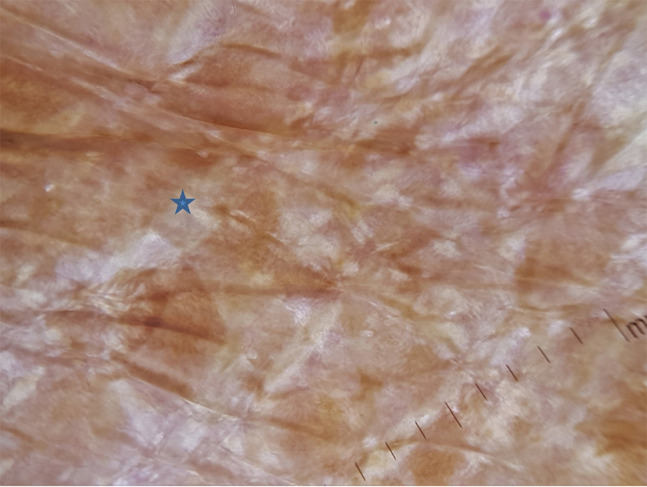
Dermoscopy of skin of dorsum of hand shows lack of eccrine gland openings, structureless white areas (blue star) suggestive of atrophy, and scarring (DermLite DL4, polarized view).

**Figure 8:**
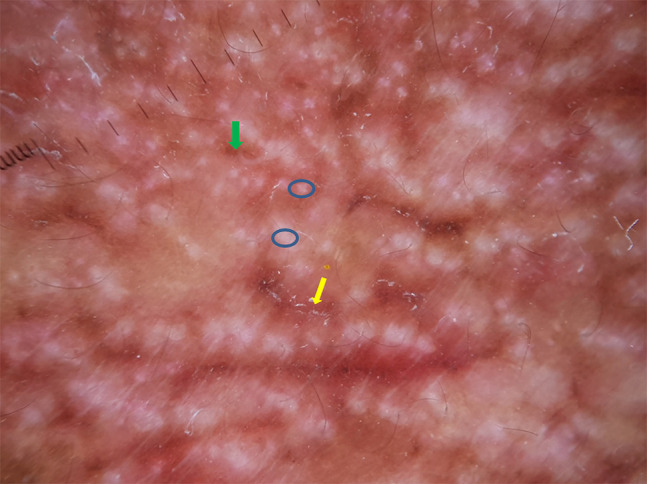
Dermoscopy of skin of fingers shows absence of dermatoglyphics.

**Figure 9:**
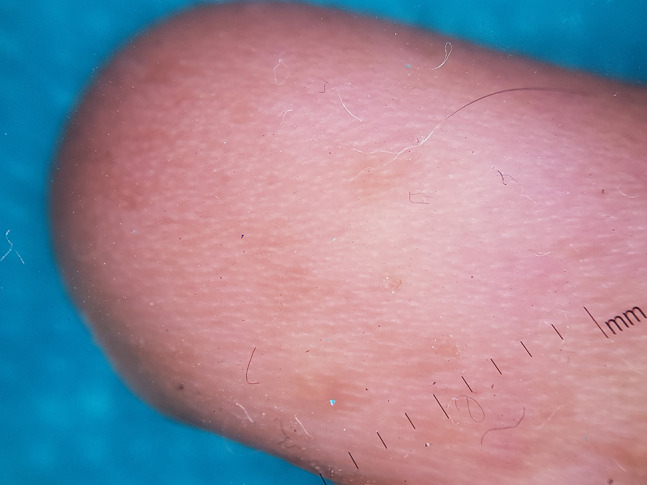
Dermoscopy of neck skin shows background erythema, white polygonal areas (blue circles), dotted vessels (yellow arrow), and uneven pigmented dots (green arrow).

**Figure 10:**
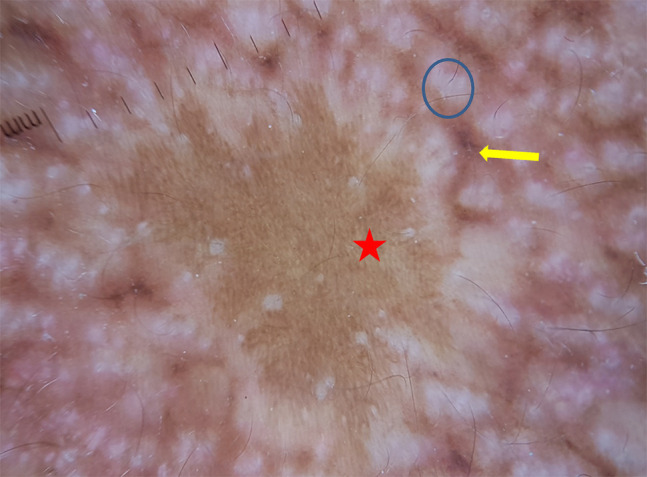
Dermoscopy of neck skin shows white polygonal areas (blue circle), pigmented dots (yellow arrow), and presence of normal skin with reticular pigment pattern (red star).

A 16-year-old boy with a history of second-degree consanguinity and similar complaints in a younger sibling presented with complaints of acral bullae in childhood, photosensitivity, thinning of skin involving dorsum of hands and feet, and redness of eyes. On examination, the patient was of average build and had normal physical and mental development. Dermatological examination revealed poikiloderma involving the neck, cigarette paper scarring of skin of dorsum of hands and feet, oral leukokeratosis, conjunctivitis, gingivitis, and palmar keratoderma (Figures 1–6). We performed dermoscopy of involved skin. Dermoscopy of skin of dorsum of hands shows absence of eccrine gland opening and reticular pigment network and presence of white structureless areas suggestive of atrophy and scarring; tips of fingers show absence of dermatoglyphic pattern; neck skin shows background erythema, dotted vessels, and pigmented dots suggestive of poikiloderma. Dermoscopy of neck skin also shows the island of normal skin with a reticulate pigmentary pattern in between affected skin, suggestive of revertant mosaicism (Figures 7–10). The rest of the dermatological and systemic examination was within normal limits. Based on history and classic clinical features, Kindler syndrome was diagnosed. Genetic testing was advised for confirmation, but the patient refused as he could not afford the test.

## Conclusions

Kindler syndrome is a rare disorder with acral bullae in infancy, poikiloderma, and photosensitivity as predominant features. Other features are eye involvement in the form of conjunctivitis, conjunctival scarring, corneal erosion, and ectropion of lower lids; oral involvement in the form of gingivitis, periodontitis, premature loss of teeth, and leukokeratosis; urethral meatal stenosis and urethral stricture, labial synechiae and vaginal stenosis, and esophageal or rectal mucosal stenosis. Waxy hyperkeratosis of palms and soles with loss of dermatoglyphics, pseudosyndactyly, and axillary freckling are other features. Patients with Kindler syndrome have a higher risk of development of squamous cell carcinoma [1]. Revertant mosaicism is a genetic phenomenon that results in spontaneous partial or complete correction of affected phenotype, characterized by the presence of a healthy patch of skin in the affected skin and can be confirmed by immunostaining of normal skin [2]. Light microscopy of Kindler syndrome shows hyperkeratosis, epidermal atrophy, and focal vacuolization of basal keratinocytes, melanophages, and colloid bodies. Electron microscopy shows the reduplication of lamina densa. The identification of the loss-of-function mutation in the *FERMT1* gene confirms the diagnosis. Our patient had classic clinical features and revertant mosaicism. Dermoscopy helped in the identification of features such as adermatoglyphia and revertant mosaicism and in documenting poikiloderma and cigarette paper scarring. Dermoscopy may not be required for confirmation of diagnosis of this rare disorder, but it may help us in delineating some subtle clinical features. To our knowledge, this is the first time dermoscopy of Kindler syndrome has been described.
